# Supplementary material for: The causal relationship between the human gut microbiota and pyogenic arthritis: a Mendelian randomization study
Source: Front Cell Infect Microbiol. 2024 Nov 26;14:1452480. doi: 10.3389/fcimb.2024.1452480 (PMC11629706; doi:10.3389/fcimb.2024.1452480)
Supplement: Supplementary file 1 [file DataSheet1.zip › mendelian test/ebi-a-GCST90027515.csv_forest.pdf]

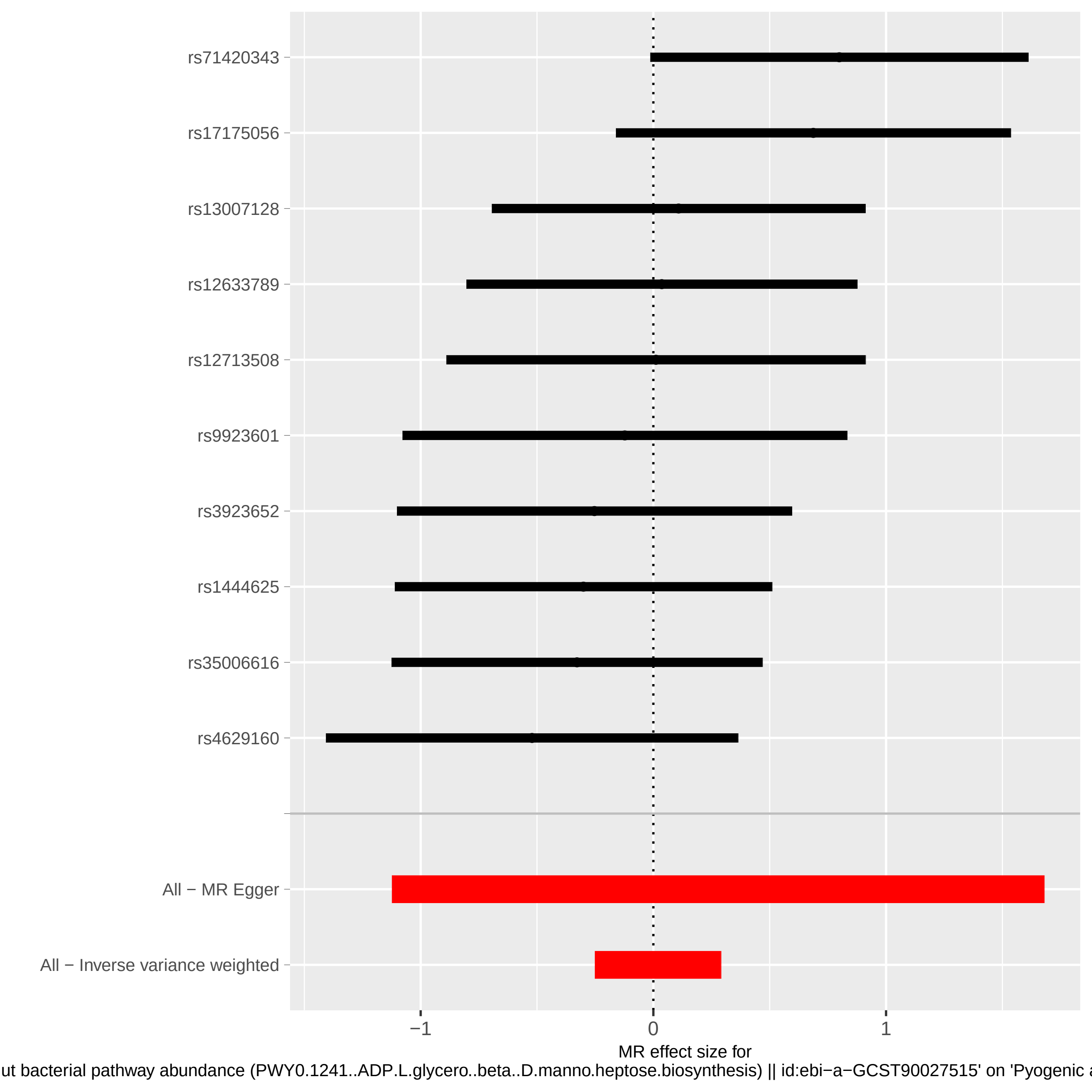

rs71420343
rs17175056
rs13007128
rs12633789
rs12713508
rs9923601
rs3923652
rs1444625
rs35006616
rs4629160
All − MR Egger
All − Inverse variance weighted
−1
0
1
MR effect size for
ut bacterial pathway abundance (PWY0.1241..ADP.L.glycero..beta..D.manno.heptose.biosynthesis) || id:ebi−a−GCST90027515' on 'Pyogenic a
